# Theoretical structural study of van der Waals complexes between oxazole and atmospheric gases CO _2_ and N _2_ for capture applications.

**DOI:** 10.12688/openreseurope.18925.2

**Published:** 2025-07-29

**Authors:** A. Belasri, F. Tahiri, O. Douass, N. Inostroza-Pino, M. Belmouden, H. Bahmann, M. Mogren Al-Mogren, M. L. Senent, S. Dalbouha

**Affiliations:** 1 Laboratory of Organic and Physical Chemistry, Research Team: Molecular Modeling, Materials, and Environment, Department of Chemistry, Faculty of Sciences Agadir, Ibn Zohr University, Agadir, Morocco, B.P. 8106, Morocco; 2 Optics, Material and Systems Team, , Faculty of Sciences, Abdelmalek Essâadi University, B.P. 2121, M’Hannech II, 93030 Tétouan, Morocco; 3 Facultad de Ingeniería, Núcleo de Astroquímica & Astrofísica, Universidad Autónoma de Chile, Av. Pedro de Valdivia 425, Providencia, Santiago, Chile; 4 Physical and Theoretical Chemistry, University of Wuppertal, Wuppertal, Germany; 5 Department of Chemistry, College of Sciences, King Saud University, P.O. Box 2455, Riyadh, 11451, Saudi Arabia; 6 Departamento de Química y Física, Instituto de Estructura de la Materia, Madrid, Unidad Asociada GIFMAN, CSIC-UHU, Spain

**Keywords:** Adsorption, CO2 capture, N2, atmosphere, ab initio, MOF’s.

## Abstract

**Background:**

The objective of this study is to explore the potential of oxazole (C _3_H _3_NO), a fascinating heterocyclic compound naturally present, which is a potential ligand in the construction of Metal-Organic Frameworks (MOFs) for the selective capture of CO _2_ in a nitrogen-rich atmosphere, using both molecular and solid-state simulation techniques.

**Methods:**

This study investigates the equilibrium structures and binding energies of van der Waals aggregates formed by an oxazole molecule with nonpolar molecules such as CO _2_ and N _2_, considering both two-body systems (oxazole@CO _2_ and oxazole@N _2_) and three-body systems (oxazole@CO _2_@N _2_ and oxazole-CO _2_/N _2_@Au _6_/Cu _6_/Zn _3_O _3_). Molecular computations for these systems are conducted using ab initio calculations at the MP2/aug-cc-pVXZ level of theory, where X = (D, T). Additionally, solid-state simulations analyze the adsorption behaviors and energies of oxazole@CO _2_ and oxazole@N _2_ on metallic surfaces:Au, Cu and ZnO(111) through Monte Carlo methods.

**Results:**

We find that the oxazole exhibits more adsorption selectivity for CO _2_ than for N _2_. Adding a second gas to the most stable complexes, oxazole@CO _2_ and oxazole@N _2_, the oxazole capture ability does not vary. On the contrary, it strengthens the adsorption energy of three-body complexes compared to two-body complexes. The addition of metallic clusters (Au _6_, Cu _6_, Zn _3_O _3_) and metallic surfaces (Au, Cu, ZnO) enhances the adsorption capacity, where Cu _6_ is particularly efficient. Both ZnO and Cu surfaces offer significant adsorption advantages while remaining economically feasible.

**Conclusions:**

This study demonstrates that oxazole exhibits a strong selectivity for CO _2_ over N _2_, with the addition of metallic clusters and surfaces significantly enhancing its adsorption capacity. These findings highlight the potential of oxazole-based materials for effective gas capture and separation, with positive implications for environmental sustainability.

## Plain language summary

This research project focuses on gas capture and separation, especially for molecules like carbon dioxide (CO _2_) and nitrogen (N _2_), which are crucial in both industrial and environmental contexts. Metal-organic frameworks (MOFs) show promise for efficient CO _2_ capture due to their adjustable structures and large surface areas. However, the use of oxazole, a specific molecule, for CO _2_ capture in these structures has been little explored. This study examines how oxazole interacts with CO _2_ and N _2_ molecules, forming aggregates bound by van der Waals forces (a type of molecular attraction). Results show that oxazole favors binding with CO _2_ over nitrogen, making it potentially useful for gas separation. By adding metallic clusters, such as gold, copper, or zinc oxide, the adsorption capacity of oxazole further improves, especially with copper, while remaining affordable.

This study highlights oxazole's potential for effective gas capture, contributing to more sustainable and environmentally friendly technologies.

## Introduction

The exploration of carbon dioxide capture and separation from gas mixtures by adsorption based on porous materials searching for low-cost and high efficiency, has been a focal point in research ^1– 7^, aiming to address environmental concerns and enhance industrial processes ^5, 6^. Gas adsorption and separation, particularly involving quadrupolar species such as carbon dioxide (CO _2_) and nitrogen (N _2_), have gained considerable interest due to their importance in industrial and environmental contexts. Metal-organic frameworks (MOFs) are porous crystalline materials formed by organic structures that act as ligands ^8^ connected by metal ions or clusters ^9, 10^. They have been considered to be efficient for CO _2_ capture tools owing to their adjustable and tunable structures ^2^, extensive surfaces ^11, 12^, large surface areas ^13, 14^, customizable functionalities ^15, 16^, and high mechanical and thermal stabilities ^17^. Specific organic ligands, such as heterocycles like carboxylic acids ^18, 19^, amines ^20^, imidazoles ^21– 25^, triazoles ^26^, pyridines ^27^, benzenes ^28^, naphthalenes ^29^, benzimidazoles ^30^, etc., enable the regulation of (MOFs) properties, thereby enhancing their capability for selective adsorption and thermal stability ^31^, making them particularly suitable for gas adsorption and separation processes ^32^. (MOFs) based on imidazole have gained interest due to the ability to design various types of architectures such as rigid, flexible, interpenetrating, etc., to influence the size of cavity pores and different types of supramolecular interactions within the pore ^33, 34^.

In our previous study ^21– 24^, we highlighted the significant ability of imidazole to form van der Waals complexes with several greenhouse gases, including CO _2_, CH _4_, and SF _6_, as well as with pollutants such as SO _2_ and NH _3_ and gas such as CO. This interaction is particularly relevant for selectively capturing CO _2_ from mixtures containing these gases. Although oxazole (C _3_H _3_NO) is a fascinating heterocyclic compound naturally present ^35^, its capacity as a ligand in the construction of CO _2_ capturing (MOFs) has not been thoroughly investigated. This provides an opportunity for further research aimed to valorize the properties of oxazole in developing efficient (MOFs) for CO _2_ capture, contributing to advancements in environmental sustainability and gas separation technologies. Our goal is the theoretical characterization of van der Waals aggregates formed with one oxazole molecule and nonpolar molecules such as carbon dioxide (CO _2_) and nitrogen (N _2_). CO _2_ is known to have significant environmental impacts on global weather patterns and human life ^36^. Anthropogenic emissions of N _2_ can indirectly lead to an increase in NO _x_ levels in the atmosphere. When N _2_ reacts with atmospheric oxygen, it converts into nitrogen oxides (NO _x_) such as NO and NO _2_. These NO _x_ compounds have consequences on air quality, human health, and the environment, contributing to smog formation, acid rain, and causing harmful effects on respiratory systems and lung tissues ^37^.

In this work, we perform a systematic evaluation of ligands containing both nitrogen and oxygen for their interaction energies with CO _2_ and N _2_ by using both molecular and solid-state simulation techniques. In the first approach, we use the possible accurate ab initio methods to calculate the binding energies (BEs) of the two body systems (CO _2_-Oxazole-BEs & N _2_-Oxazole-BEs). The analysis of the ternary systems (CO _2_-oxazole-N _2_-BEs) and (oxazole-CO _2_/N _2_@Au _6_/Cu _6_/Zn _3_O _3_) using the same method was conducted to understand the interactions among CO _2_, oxazole, and N _2_ within these systems, to examine their influence on CO _2_ separation and capture strategies in gas mixtures, and predict the capacity of new materials to selectively capture and store CO _2_ over N _2_ in N _2_-rich atmospheres. Indeed, the presence of N _2_ can significantly influence CO _2_ capture performance in (MOFs), taking into account their common presence in atmospheric and industrial environments, emphasizing the crucial role of computational methods in designing nanomaterials for carbon capture technologies.

On the other hand, the Monte Carlo solid-state simulations were employed to investigate the interactions between oxazole and the (111) surfaces of Au, Cu, and ZnO. These materials were selected for their unique electronic properties and practical relevance. Gold (Au) is frequently used as a reference due to its stability and well-characterized surface chemistry ^38^, Copper (Cu) is chosen for its economic viability and its proven effectiveness in enhancing adsorption processes ^39^. While zinc oxide ZnO was included in this study for its unique semiconducting, piezoelectric, and photoelectric properties and high surface area ^40^.

### Computational details

All the electronic computations were performed using both the Gaussian 09 Revision C.01 software ^41^ and the Material Studio (BIOVIA, 2020) program ^42^. An appropriate free alternative software for this purpose is GAMESS (US), a powerful tool for electronic structure calculations that supports HF, DFT, MP2, and other methods, and ORCA, a versatile quantum chemistry program renowned for its computational efficiency and support for DFT, HF, and post-HF techniques.

 We have combined the most accurate possible ab initio method, and Monte Carlo solid-state simulations ^43^, to investigate the interaction between CO _2_ and N _2_ and oxazole (Oxa), either isolated, attached to gold, copper and ZnO cluster or surface.

The study employed the second-order Møller–Plesset theory (MP2) ^44, 45^ within the Gaussian software to compute the equilibrium structures and spectroscopic properties of both isolated species and complexes included two-body systems like oxazole@CO _2_ and oxazole@N _2_. Additionally, the three-body systems such as oxazole@CO _2_@N _2_ and oxazole-CO _2_/N _2_@Au _6_/Cu _6_/Zn _3_O _3_ were investigated. For an accurate depiction of small systems, in these computations, the atoms were described using Dunning and co-workers’ aug-cc-pVXZ basis set (abbreviated as AVXZ, where X=D, T) ^46^, augmented with diffuse atomic orbitals to capture long-range interactions. Specifically, AVTZ was employed for oxazole@CO _2_ and oxazole@N _2_, while AVDZ was used for oxazole@CO _2_@N _2_ and oxazole@CO _2_/N _2_@ cluster complexes. 

Monte Carlo (MC) simulation ^47^ is a widely used computational method for investigating molecular adsorption on surfaces, particularly for estimating adsorption energies ( *E*_ads_) and modeling adsorption isotherms. Based on the Metropolis algorithm ^48^, it explores configurational space by evaluating randomly generated molecular states according to energy changes and thermodynamic probability. In this study, the Adsorption Locator module, implemented in Materials Studio 2020 (BIOVIA) ^42^, was employed in conjunction with the COMPASS II force field ^49^ to examine the adsorption energies of oxazole@N _2_, and oxazole@CO _2_ on gold, copper, and zinc oxide surfaces. Atomistic grand canonical Monte Carlo (GCMC) simulations were performed to estimate the adsorption isotherms to estimate the adsorption isotherms of The adsorption isotherms of oxazole@CO _2_ and oxazole@N _2_ on Au (111), Cu (111), and ZnO (111) surface.

As a free alternative to Material Studio, I recommend the open-source software Quantum ESPRESSO and CP2K. The first program, Quantum ESPRESSO, is a comprehensive suite for quantum simulations, ideal for materials modeling and the simulation of solids, liquids, and nanostructures.

https://www.quantum-espresso.org/

The second program, CP2K, is also free and open-source software, widely used for molecular dynamics simulations and quantum chemistry calculations, particularly for systems with large numbers of atoms.

https://www.cp2k.org/

**Figure 5.**
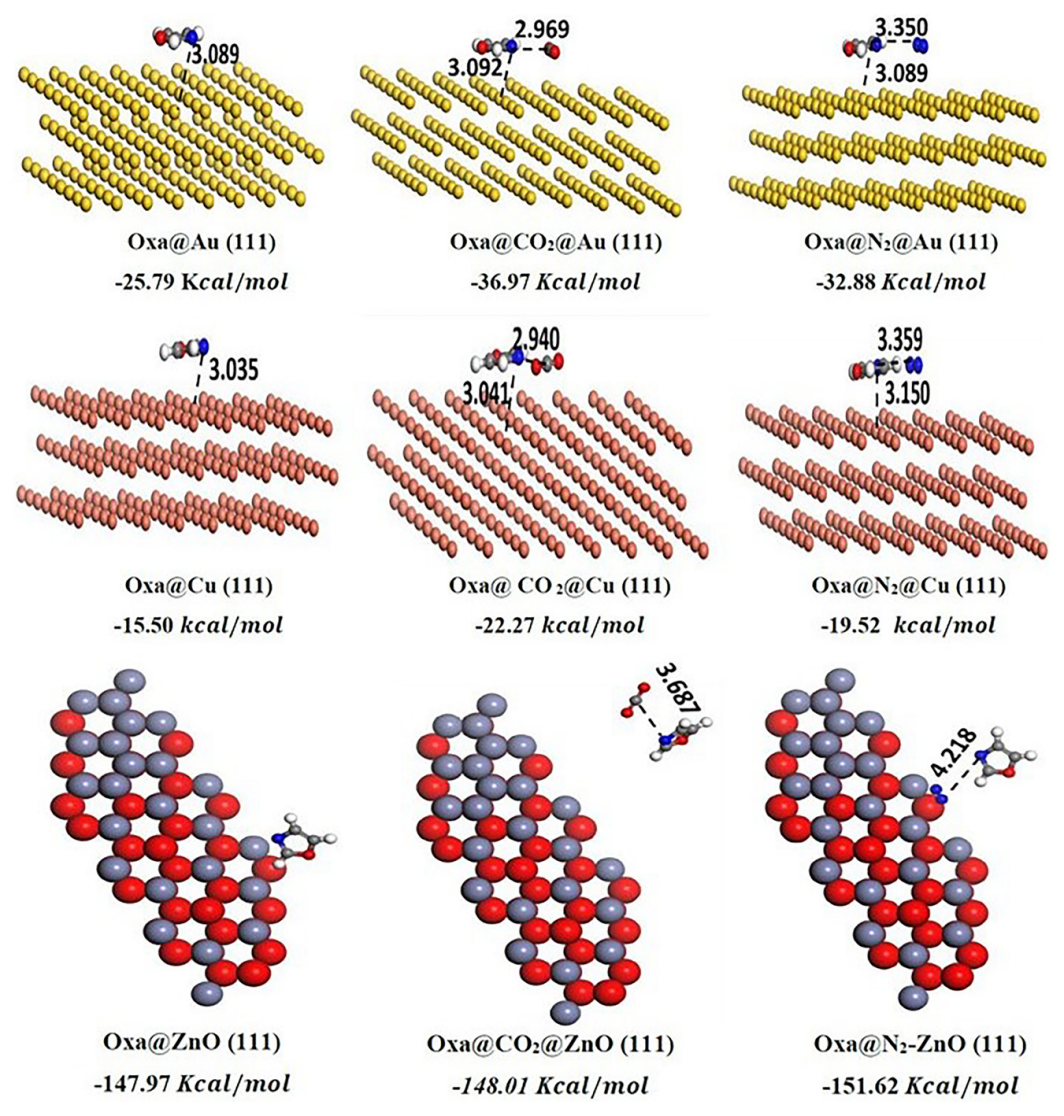
Optimized structures of Oxa@ Au (111) /Cu (111) and ZnO (111) and of CO _2_/N _2_–Oxa@ Au (111)/ Cu (111) and ZnO (111) complexes, derived from the MC simulation.

The Au, Cu, and ZnO crystal structures have been imported from the Materials Studio database. Surfaces were built by clicking 'Build', then 'Surface', and then 'Cleave Surface'. In the following menu, the cleaving plane (1 1 1) was chosen, and then afterward, the 'Symmetry' option was used to construct a supercell. All the surface structures were prepared with three layers, as illustrated in Figure 5.

The structures of oxazole, oxazole@N _2_, and oxazole@CO _2_ were well optimized following the then possible lowest energy using the COMPASS II force field as implemented in the Forcite Module. This same force field was used in the Monte Carlo simulations. The simulations were done utilizing the Adsorption Locator Module in the Materials Studio 2020 package from BIOVIA. These simulations involved 10 cycles of 10,000 steps each, providing insights into adsorption processes relevant to catalysis, materials science, and environmental applications.

Therefore, a detailed investigation of adsorption behaviors and energies of the fixed molecules on the chosen surfaces provides valuable insight into their eventual interactions and stability.

The minimum character of all the optimized structures is evidenced by the presence of all positive harmonic frequencies, indicating that the system is at its energy minimum, reflecting a stable and optimized configuration. The binding energy (BE) between oxazole and CO _2_ or N _2_ was evaluated for optimized geometries using Second-order Møller-Plesset perturbation theory (MP2) in connection with aug-cc-pVTZ basis set (denoted in this paper by AVTZ). The binding energies (BEs) were calculated using the following energy expression:

BE= E _Complex_ - (E _oxazole_+ E _gaz_)

where: *BE* is the binding energy, *E _complex_* is the total energy of the oxazole-gas complex, *E _Oxazole_* is the total energy of the isolated oxazole molecule, and *E _gaz_* is the total energy of the isolated gas molecule (CO _2_ or N _2_). For a more accurate estimation of the interaction energy, the basis set superposition error (BSSE) was corrected using the Boys and Bernardi method ^50^. Additionally, the zero-point vibrational energy correction (ZPVE) was also taken into account for all geometries ^51^.

For the complexes between oxazole and CO _2_ or N _2_ @ cluster (Au _6_, Cu _6_ or Zn _3_O _3_) we employed the MP2/AVDZ/LanL2DZ ^52^, and for the adsorption of CO _2_ and N _2_ on Au, Cu, and ZnO (111) surfaces, we used the Monte Carlo computational technique.

The binding energies (BEs) were calculated using the following energy expression:

BE = E _Complex_ - (E _Oxa@CO2/N2_+ E _Cluster/Surface_)

where: *BE* is the binding energy *E _complex_*, is the total energy of the Oxa CO _2_/N _2_@ cluster (cluster = Au _6_/Cu _6_ or Zn _3_O _3_) or Surface Au, Cu or ZnO (111) complexes, *E*_Oxa@CO _2_/N _2__ is the total energy of the complex Oxazole+CO _2_ or Oxazole+N _2_, and *E _Cluster/Surface_* is the total energy of the isolated gas cluster (Au _6_, Cu _6_ or Zn _3_O _3_) or Surface Au(111), Cu (111) or ZnO (111). For a more accurate estimation of the interaction energy of Oxazole CO _2_/N _2_@ cluster (cluster= Au _6_/Cu _6_ or Zn _3_O _3_) the zero-point vibrational energy correction (ZPVE) was also taken into account for all geometries.

## Results and discussions

### Equilibrium structure of the complexes

**Figure 1.**
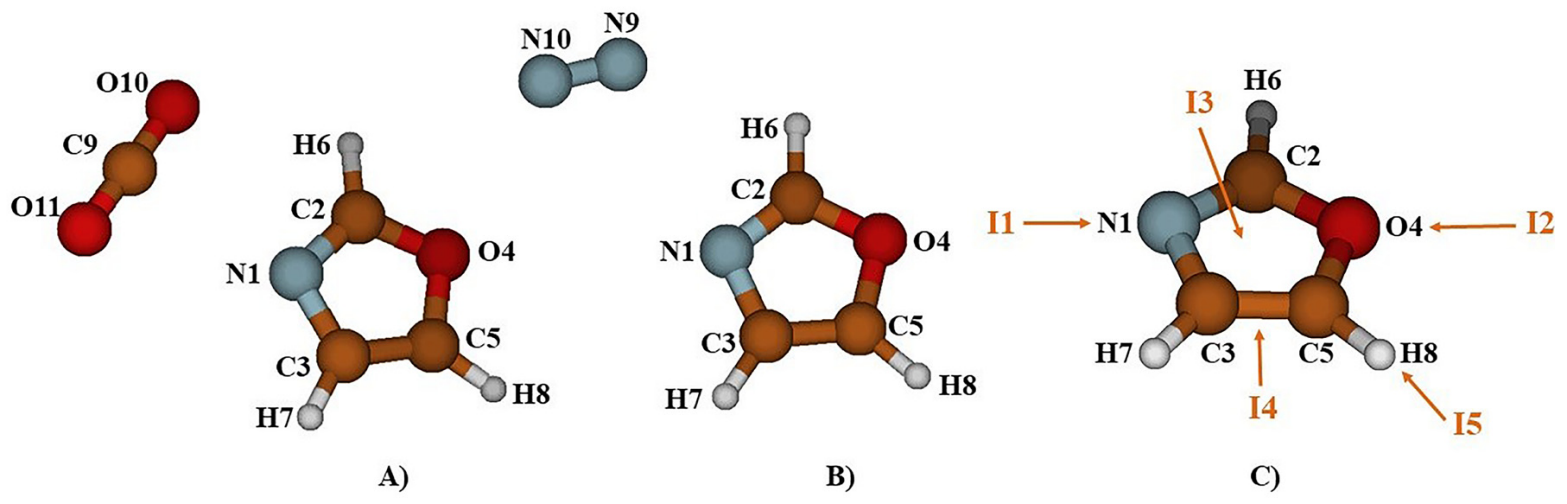
The atom labeling of the complex Oxazole + CO _2_ ( **A**), Oxazole + N _2_ ( **B**) and Oxazole and the five principal complexation pathways, I1, I2, I3, I4, and I5 ( **C**).

To examine the molecular-level adsorption of CO _2_ and N _2_ on oxazole, we first located the CO _2_ and N _2_ molecules at the N1 site of oxazole (refer to Figure 1) and then we oriented them around it to explore all possible equilibrium structures. It's worth noting that the number of stable structures increases the likelihood of gas capture. Despite multiple attempts, the optimization results at the MP2/AVTZ level consistently converged toward one of the structures presented in Figure 1A and 1B, which characterize the complexes formed by oxazole with CO _2_ and N _2_.

**Figure 2.**
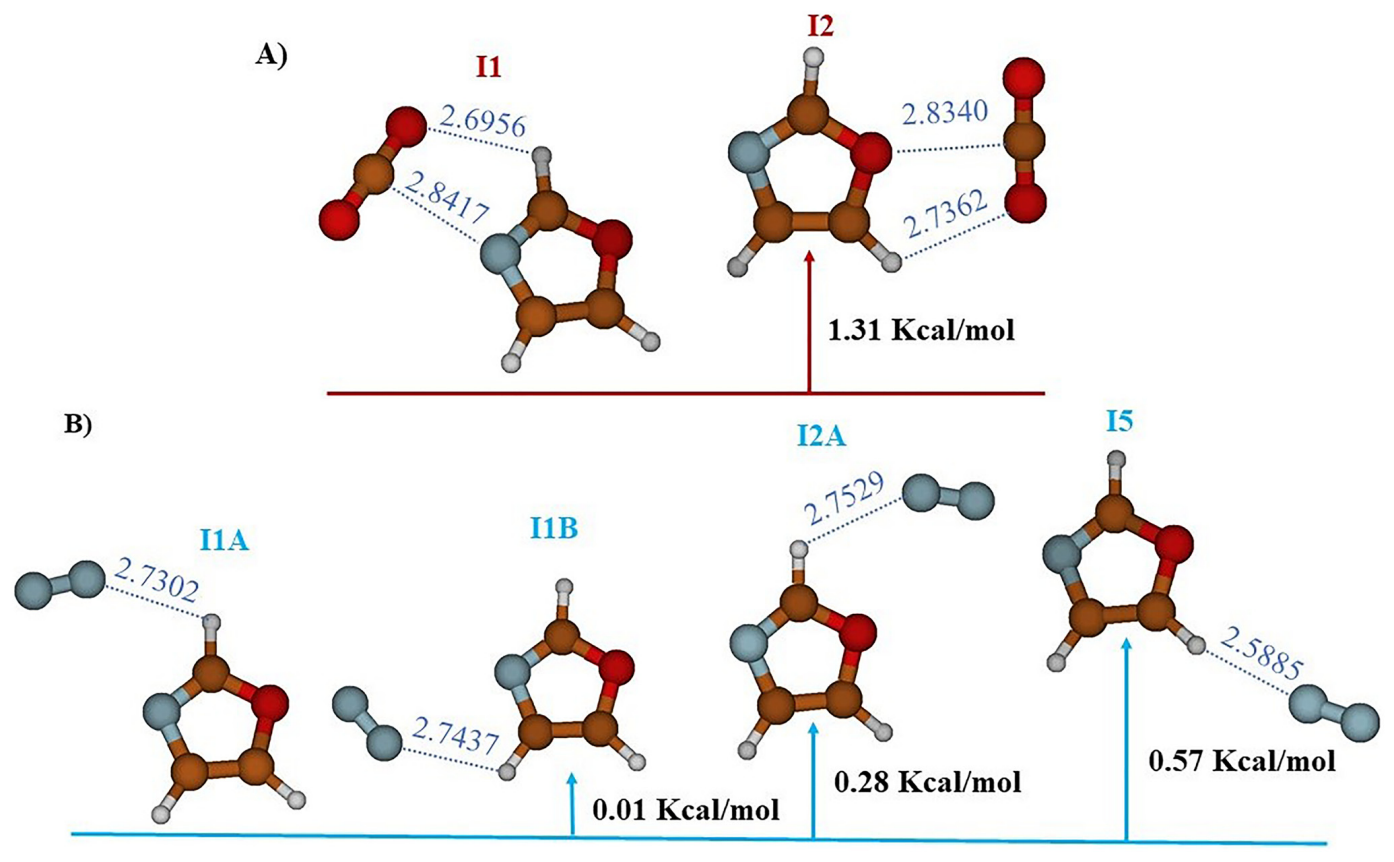
The equilibrium geometries and the MP2/AVTZ relative energies of the oxazole +CO _2_ ( **A**) and oxazole + N _2_ ( **B**) Van der Waals complexes.

The study carried out at the MP2/AVTZ level on the Oxazole- CO _2_ complex reveals the existence of two stable structures labeled I1 and I2 (cf. Figure 2A, Table 1). In structure I1 (E _r_ = 0.0 kcal/mol), identified as the global minimum, the bond is established through an interaction between the electron donor-acceptor (EDA) sites of CO _2_ and oxazole (RO10-H6 = 2.6956 Å and RC9-N1 = 2.8418 Å). However, in structure I2 (E _r_ = 1.31 kcal/mol), the bond is maintained through a donor-acceptor electron exchange (EDA), where the oxygen atom of CO _2_ interacts with H8 of oxazole (RO10H8 = 2.7362 Å) and the carbon atom of CO _2_ interacts with the oxygen of oxazole (RC9-O4 = 2.8340 Å).

**Table 1.** Total electronic energies (E, in a.u.), relative energies (E _r_, in kcal/mol) and structural parameters (Distances in Å; angles in degrees) of the two equilibrium structures of the Oxazole-CO _2_ complexes calculated with MP2/aug-cc-pVTZ.

Oxazole (C1)		I1 (C1)		I2 (C1)	
		$\frac{\text{ROH}}{\text{RCN}}$	$\frac{2.6956}{2.8418}$	$\frac{\text{ROH}}{\text{RCO}}$	$\frac{2.7362}{2.8340}$
E	-245.639836	E	-433.968056	E	-433.965972
		E _R_	0.0	E _R_	1.31
*C2N1*	*1.2996*	*C2N1*	*1.3011*	*C2N1*	*1.2987*
*C3N1*	*1.3862*	*C3N1*	*1.3858*	*C3N1*	*1.3869*
*O4C2*	*1.3552*	*O4C2*	*1.3531*	*O4C2*	*1.3570*
*C5C3*	*1.3596*	*C5C3*	*1.3597*	*C5C3*	*1.3593*
*H6C2*	*1.0755*	*H6C2*	*1.0757*	*H6C2*	*1.0757*
*H7C3*	*1.0752*	*H7C3*	*1.0752*	*H7C3*	*1.0752*
*H8C5*	*1.0739*	*H8N4*	*1.0739*	*H8N4*	*1.0741*
*C3N1C2*	*104.0*	*C3N1C2*	*104.2*	*C3N1C2*	*104.1*
*O4C2N1*	*114.7*	*O4C2N1*	*114.5*	*O4C2N1*	*114.6*
*C5C3N1*	*109.1*	*C5C3N1*	*109.0*	*C5C3N1*	*109.3*
*H6C2N1*	*128.6*	*H6C2N1*	*128.2*	*H6C2N1*	*128.8*
*H7C3N1*	*122.1*	*H7C3N1*	*122.1*	*H7C3N1*	*122.0*
*H8C5O4*	*116.7*	*H8C5O4*	*116.8*	*H8C5O4*	*116.6*
CO _2_					
E	-188.321641				
*CO*	*1.1702*	*O10C9*	*1.1718*	*O10C9*	*1.1709*
		*O11C9*	*1.1686*	*O11C9*	*1.1690*
*<OCO*	*180.0*	*<OCO*	*177.6*	*<OCO*	*179.3*
		*O10H6*	*2.6956*	*O10H8*	*2.7362*
		*O10H6C2*	*104.2*	*O10H8C5*	*111.2*
		*C9O10H6*	*114.7*	*C9O10H8*	*113.6*
		*C9N1*	*2.8418*	*C9O4*	*2.8340*
		*C9N1C2*	*106.8*	*C9O4C2*	*142.8*
		*O10C9N1*	*86.1*	*O10C9O4*	*85.8*

Additionally, the study of the complex between N _2_ and oxazole, still at the MP2/AVTZ level, revealed the existence of four adsorption sites as outlined in Figure 2B and Table 3. These sites are labeled as follows: I1A (E _r_ = 0.0 kcal/mol), I1B (E _r_ = 0.01 kcal/mol), I2A (E _r_ = 0.28 kcal/mol), and I5 (E _r_ = 0.57 kcal/mol). These distinct structures depict various stable configurations of the N _2_-oxazole complex. Predominantly, the key interactions observed in the stable structures occur where hydrogen bonds link N _2_ to oxazole. Additionally, the equilibrium distances within the van der Waals systems RN9Hx (X=6,7,8) exhibit the following variations: RN9H6 = 2.7302 Å for I1A, RN9H7 = 2.7437 Å for I1B, RN9H6 = 2.7529 Å for I2A, and RN9H8 = 2.5885 Å for I5.

Table 1 and Table 3 present the structural parameters at the MP2/AVTZ level, while Table 2 and Table 4 provide the MP2/AVTZ harmonic frequencies for each examined complex. The parameters of the van der Waals aggregates are compared to those of the corresponding isolated components.

**Table 2.** Harmonic fundamental frequencies (ω, in *cm ^-1^*) of CO _2_, oxazole, and the two equilibrium geometries of the oxazole-CO _2_ complexes calculated at the MP2/AVTZ level of theory.

ω								
	*Oxazole*		*CO _2_*		*I1*		*I2*	
ω _1_	a	3330			a	3331	a	3330
ω _2_		3311				3312		3309
ω _3_		3299				3300		3299
ω _4_			σ _u_	2402		2402		2404
ω _5_		1546				1546		1547
ω _6_		1508				1506		1507
ω _7_		1355				1355		1354
ω _8_			σ _g_	1326		1327		1328
ω _9_		1272				1269		1270
ω _10_		1185				1189		1182
ω _11_		1141				1144		1138
ω _12_		1109				1111		1108
ω _13_		1086				1086		1082
ω _14_		915				917		917
ω _15_		905				909		904
ω _16_		864				865		868
ω _17_		827				836		827
ω _18_		763				766		771
ω _19_		665				667		664
ω _20_			π	659		662		660
ω _21_			π	659		637		651
ω _22_		628				628		626
ω _23_						132		100
ω _24_						88		80
ω _25_						52		39
ω _26_						44		38
ω _27_						29		21

**Table 3.** Total electronic energies (E, in a.u.), relative energies (E _r_, in kcal/mol) and structural parameters (Distances in Å; angles in degrees) of the four equilibrium structures of the Oxazole-N2 complex calculated with MP2/aug-cc-pVTZ.

Oxazole (C1)		I1A (C1)		I1B (C1)		I2A (C1)		I5 (C1)	
		**R _NH_**	2.7302	**R _NH_**	2.7437	**R _NH_**	2.7529	**R _NH_**	2.5885
E	-245.639836	E	-355.007379	E	-355.007363	E	-355.006923	E	-355.006474
		**E _R_**	**0.0**	**E _R_**	**0.01**	**E _R_**	**0.28**	**E _R_**	**0.57**
*C2N1*	2.9962	*C2N1*	1.3003	*C2N1*	1.30006	*C2N1*	1.2997	*C2N1*	1.2998
*C3N1*	1.38622	*C3N1*	1.3860	*C3N1*	1.3867	*C3N1*	1.3859	*C3N1*	1.3863
*O4C2*	1.35519	*O4C2*	1.3549	*O4C2*	1.3547	*O4C2*	1.3565	*O4C2*	1.3550
*C5C3*	1.35957	*C5C3*	1.3597	*C5C3*	1.3597	*C5C3*	1.3597	*C5C3*	1.3599
*H6C2*	1.07554	*H6C2*	1.0754	*H6C2*	1.0756	*H6C2*	1.0752	*H6C2*	1.0755
*H7C3*	1.07521	*H7C3*	1.0752	*H7C3*	1.0751	*H7C3*	1.0752	*H7C3*	1.0752
*H8C5*	1.07390	*H8C5*	1.0739	*H8C5*	1.07388	*H8C5*	1.0733	*H8C5*	1.0738
*C3N1C2*	103.99	*C3N1C2*	104.0	*C3N1C2*	104.0	*C3N1C2*	104.0	*C3N1C2*	104.0
*O4C2N1*	114.70	*O4C2N1*	114.7	*O4C2N1*	114.7	*O4C2N1*	114.6	*O4C2N1*	114.7
*C5C3N1*	109.13	*C5C3N1*	109.1	*C5C3N1*	109.1	*C5C3N1*	109.1	*C5C3N1*	109.2
*H6C2N1*	128.59	*H6C2N1*	128.4	*H6C2N1*	128.6	*H6C2N1*	128.8	*H6C2N1*	128.7
*H7C3N1*	122.09	*H7C3N1*	122.1	*H7C3N1*	122.0	*H7C3N1*	122.1	*H7C3N1*	122.0
*H8C5O4*	116.75	*H8C5O4*	116.9	*H8C5O4*	116.8	*H8C5O4*	116.8	*H8C5O4*	116.7
*N _2_*									
E	-109.364800								
*NN*	*1.11401*	NN	1.11387		1.11408		1.11396		1.11401
		*N9H6*	2.7302	N9H7	2.7437	N9H6	2.7529	N9H8	2.5885
		*N9H6C2*	109.2	N9H7C3	113.3	N9H6C2	114.9	N9H8C5	167.7
		*N10N9H6*	147.3	N10N9H7	144.1	N10N9H6	114.9	N10N9H8	172.1

**Table 4.** Harmonic fundamental frequencies (ω, in *cm ^-1^*) of N _2_, oxazole and the four equilibrium geometries of the oxazole-N _2_ complexes calculated at the MP2/AVTZ level.

Ω												
	*Oxazole*		*N _2_*		*I1A*		*I1B*		*I2A*		*I5*	
ω _1_	a	3330			a	3330	a	3330	a	3329	a	3336
ω _2_		3310				3314		3310		3316		3310
ω _3_		3298				3298		3301		3298		3299
ω _4_			σ _g_	2187		2186		2185		2186		2186
ω _5_		1545				1544		1545		1545		1546
ω _6_		1507				1506		1507		1506		1508
ω _7_		1355				1354		1354		1355		1356
ω _8_		1271				1271		1271		1271		1273
ω _9_		1185				1186		1186		1182		1186
ω _10_		1141				1142		1140		1141		1141
ω _11_		1109				1109		1110		1108		1113
ω _12_		1085				1086		1086		1085		1085
ω _13_		915				914		915		914		915
ω _14_		904				905		904		904		905
ω _15_		863				864		870		865		866
ω _16_		826				834		827		833		827
ω _17_		763				765		764		763		784
ω _18_		664				666		665		665		665
ω _19_		627				628		628		627		630
ω _20_						84		82		77		58
ω _21_						**63**		**58**		**54**		**57**
ω _22_						46		46		42		49
ω _23_						40		43		26		22
ω _24_						21		20		13		9

During the formation of weak intramolecular bonds, the structure of the oxazole ring and N _2_ undergoes minimal changes (see Table 1 and Table 3). However, a significant deformation is observed in the CO _2_ molecule, resulting in the loss of its symmetry. This deformation is particularly pronounced in the oxazole-CO _2_ complexes I1 and I2 (see Table 1), where the C-O bond in CO _2_ tends to shorten (O11C9: 1.1686 Å for I1, 1.1690 Å for I2), while the distance O10C9 is elongated (1.1718 Å for I1, 1.1709 Å for I2), compared to free CO _2_ (CO=1.1702 Å). Additionally, the <OCO angle is bent away from the linear configuration of a free CO _2_ molecule, measuring 177.6 degrees for I1 and 179.3 degrees for I2, respectively. This deformation is attributed to the formation of the van der Waals complexes between CO _2_ and oxazole, leading to electron redistribution and strengthening of the C-O bond.

Harmonic frequencies were computed to ensure minimal energy for all structures. Notably, in Table 2 and Table 4, certain low frequencies (5 frequencies for Oxazole-CO _2_, ω _23_-ω _27_, and for Oxazole-N _2_, ω _20_-ω _24_) emerge, which were not observed for the monomers. This situation allows for the use of a Born-Oppenheimer-type approach, which distinguishes between intramolecular and intermolecular vibrations. Intermolecular vibrations more closely resemble hindered rotations or tunneling motions. Generally, there is a strong coupling between different intermolecular degrees of freedom. The deformation vibrations of O–C–O in complexes I1 and I2 occur at frequencies of 637 cm ^-1^ and 651 cm ^-1^, respectively. These modes experience significant frequency shifts compared to the CO _2_ monomer, approximately 8 cm ^−1^ for complex I2 an 22 cm ^-1^ for complex I1. Furthermore, the symmetric and antisymmetric intermolecular O-C stretching modes with frequencies ω _8_ = 1328 cm ^-1^ and ω _4_ = 2402 cm ^-1^ for complex I2 have shifted by 2 cm ^-1^ from frequencies of ω _8_ = 1326 cm ^-1^ and ω _4_ = 2404 cm ^-1 ^for the CO _2_ monomer. In the oxazole-N _2_ complex, the stretching mode of N-N is not influenced by the formation of the hydrogen bond between the N of N _2_ and the H of oxazole. Thus, the intermolecular stretching vibrations H..N–N(ω _21_) in the optimal structure are observed at frequencies of 63 cm ^−1^, 58 cm ^−1^, 54 cm ^−1^, and 57 cm ^−1^ for I1A, I1B, I2A, and I5, respectively.

### Binding energies

***Two body complexes type oxazole-CO _2_ and oxazole-N _2_***

The binding energy is crucial for understanding, predicting, and enhancing the performance of adsorbent materials in greenhouse gas and pollutant capture processes. It determines the interaction between the host and the guest, as well as the properties of the adsorbent material. A more negative adsorption energy generally indicates a stronger adsorption interaction. For this reason, the binding energies of all previously mentioned stable forms, are computed via the MP2/AVTZ approach and including the zero-point vibrational energy (ZPVE) along with as well as the Boys and Bernardi correction (BSSE), are listed in Table 5. Our calculations indicate that oxazole is energetically favored as an adsorbent for both our adsorbates although CO _2_ shows a higher capacity to bind oxazole rings. The range of CO _2_ binding energies spans from -4.13 to -2.82 kcal/mol, while for N _2_, the energies range from -1.77 to -1.19 kcal/mol. Based on the adsorption energies, the nitrogen site of the oxazole is the most stable site for CO _2_ adsorption, and Hydrogen 6 is the most stable site for N _2_ adsorption. The zero-point vibrational energy (ZPVE) correction ranges from 0.39 to 0.51 kcal/mol for the CO _2_ complexes and from 0.32 to 0.39 kcal/mol for the N _2_ complexes. On the other hand, the BSSE correction is between 0.02 and 0.09 kcal/mol for CO _2_ and 0.05 kcal/mol for N _2_.

**Table 5.** MP2/AVTZ binding energies of the oxazole complexes (E _b_, kcal/mol).

Complex	E _int_	E _int_ +BSSE	E _int_ +ZPVE	E _int_ +BSSE +ZPVE
Oxazole-CO _2_				
I1	-4.13	-4.22	-3.62	-3.71
I2	-2.82	-2.84	-2.43	-2.45
Oxazole-0N _2_				
I1A	-1.77	-1.72	-1.38	-1.33
I1B	-1.76	-1.71	-1.39	-1.37
I2A	-1.48	-1.43	-1.16	-1.11
I5	-1.19	-1.14	-0.85	-0.8

***Three body complexes type CO _2_-Oxazole-N _2_***

**Figure 3.**
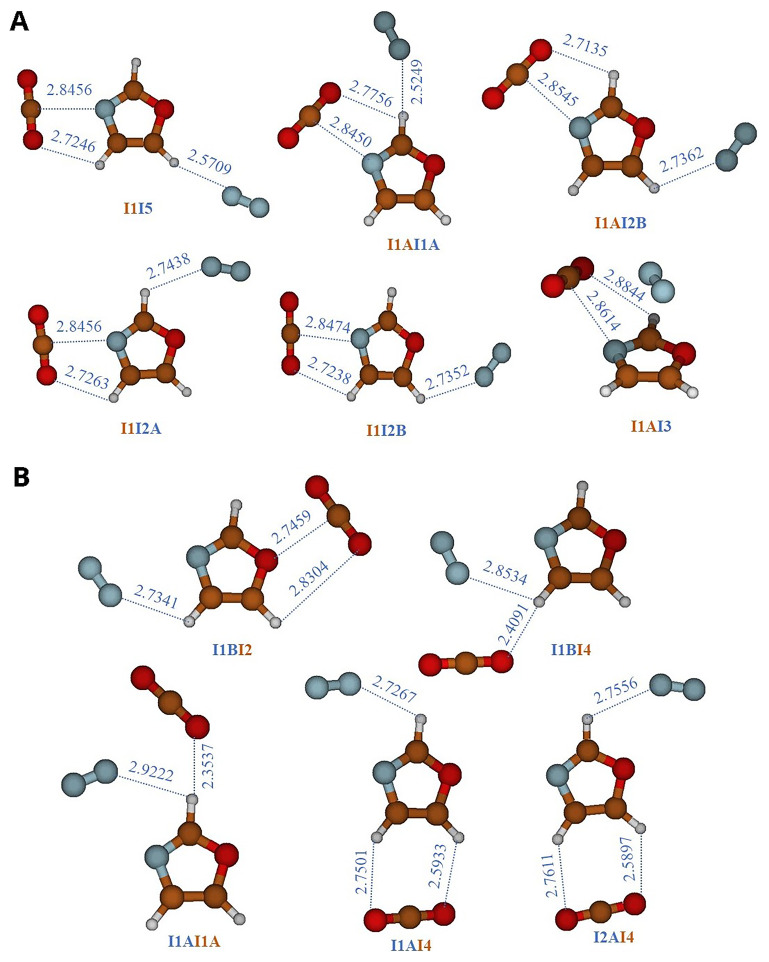
Three-body complexes composed by CO _2_-oxazole-N _2_. **A**) CO _2_ -oxazole + N _2_ complexes, they are labeled using the symbol ‘‘ In Im”.‘‘ n” denotes the location of CO _2_ and ‘‘ m” refers to the position of N _2_. **B**) N _2_ -oxazole + CO _2_ complexes, they are labeled using the symbol ‘‘ Im In”.‘‘ m ” denotes the location of N _2_ and ‘‘ n” refers to the position of CO _2_.

Since this article focuses on the CO _2_ separation properties relative to N _2_, the most abundant gas in Earth's atmosphere (78.084%) ^53^, our aim is to understand, at the molecular level, whether oxazole-based (MOFs) can simultaneously capture two different gases and how the CO _2_ capture capacity varies in the presence of N _2_, which a third body can alter the interaction between two bodies. To achieve this, we performed geometry optimizations on three-body systems, namely N _2_-oxazole-CO _2_, starting from various configurations where CO _2_ and N _2_ occupy the equilibrium positions of the two-body systems, oxazole-CO _2_ and oxazole-N _2_. We then examined the capture of the second gas. The resulting structures are presented in Table 6 and illustrated in Figure 3. In Figure 3A, the equilibrium structures of three systems are shown, obtained using the equilibrium configurations of the oxazole-CO _2_ complex and rotating N _2_ around oxazole. However, in Figure 3B, the equilibrium structures of three other systems are displayed, obtained using the equilibrium configurations of the oxazole-N _2_ complex and rotating CO _2_ around oxazole. The structures in Figure 3A are identified by the symbol "InIm," where "In" and "Im" respectively represent the main complexation pathways (I1, I2, I3, I4, and I5) of CO _2_ and N _2_ with oxazole ( Figure 1C), while in Figure 3B, they are identified by the symbol "ImIn". The binding energies in Table 6 are determined by combining the AVDZ basis set with MP2 correlation, which is an appropriate method for three-body systems. We opted for AVDZ over AVTZ due to the size of the systems, which makes the use of AVTZ impossible. These values are then compared to those of two-body complexes (oxazole+X, where X= CO _2_, N _2_) calculated using the same level of theory. It is observed that the presence of a second gas appears to be beneficial, thereby enhancing the oxazole's ability to capture both CO _2_ and N _2_ without causing competition.

**Table 6.** MP2/AVDZ binding energies of the two-body and three-body complexes (E _b_, kcal/mol).

Complex	E _int_	E _int_ +ZPVE	Complex	E _int_	E _int_ +ZPVE
Oxazole-CO _2_			Oxazole-CO _2_+N _2_		
I1	-4.48	-3.95	I1I5	-5.94	-5.13
I2	-3.13	-2.74	I1I2A	-6.18	-5.40
			I1AI2B	-6.23	-5.44
			I1AI2B	-6.57	-5.70
			I1AI1A	-6.57	-5.71
			I1AI3	-8.40	-7.31
Oxazole-N _2_			Oxazole-N _2_ +CO _2_		
I1A	-1.91	-1.51	I1AI1A	-4.85	-4.09
I1B	-1.94	-1.56	I1A I4	-3.93	-3.26
I2A	-1.61	-1.28	I1B I4	-4.81	-3.98
I5	-1.40	-1.03	I2A I4	-3.60	-2.99
			I1B I2	-5.03	-4.354

The interaction energies of the three-body systems increase and are higher than those observed in the two-body oxazole + CO _2_ complexes. In the case of the oxazole-N _2_+CO _2_ complexes, the binding energy ranges between -3.60 kcal/mol and -5.03 kcal/mol, compared to the two-body systems, oxazole+N _2_, whose binding energies are respectively between -1.40 kcal/mol and -1.94 kcal/mol. So, for the oxazole-CO _2_+N _2_ complexes, the binding energy ranges between -5.94 kcal/mol and -8.40 kcal/mol, compared to the two-body systems oxazole+CO _2_, which exhibit binding energies ranging from -3.13 kcal/mol to -4.48 kcal/mol. This is explained by the formation of an additional intramolecular bond between the molecules of the three systems (π-stacking and Van der Waals interactions), which can stabilize the systems. The inclusion of the ZPVE vibrational correction results in a decrease in the energy quantities of three systems, ranging from -0.49 kcal/mol to -1.09 kcal/mol.

***Oxa@Au _6_, Cu _6_ and Zn _3_O _3_ and CO _2_/N _2_–Oxa@Au _6_, Cu _6_ and Zn _3_O _3_***

**Figure 4.**
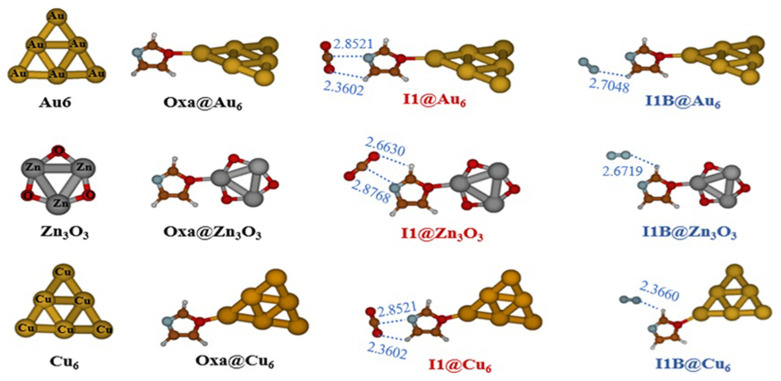
Optimized structures of Oxa@Au _6_, Cu _6_ and Zn _3_O _3_ and of CO _2_/N _2_Oxa@ Au _6_,Cu _6_ and Zn _3_O _3_ complexes at MP2/ AVDZ/LanL2DZ level of theory.

Recently, various technologies have emerged for CO _2_ capture, utilizing methods such as chemisorption and physisorption, due to the development of nanodevices such as metal nanoclusters and metal oxide nanoclusters ^54^. These nanocomposite materials have found wide-ranging applications, including energy storage ^55^, drug delivery ^56^, and the adsorptive removal of diverse pollutants from contaminated environments ^57^. These applications benefit from the materials' high stability, ease of synthesis, and ability to be modulated into various shapes and sizes. This section focuses on investigating the mechanisms of adsorption and activation of CO _2_ and N _2_ by metal clusters, Au _6_ and Cu _6_, as well as on the metal oxide Zn _3_O _3_. To bolster the CO _2_ and N _2_ adsorption capabilities, this choice of clusters was motivated by the tendency of clusters with an even number of particles to demonstrate higher stability compared to their odd-sized counterparts, along with possessing a larger HOMO-LUMO gap ^58– 60^. Moreover, these clusters exhibit a rapid increase in binding energies with increasing cluster size. Notably, these nanoparticles possess the ability to bind with various biological or medicinal molecules containing relevant heteroatoms like nitrogen and oxygen, all while exhibiting low toxicity ^61^. The CO _2_/N _2_–Oxa@Au _6_, Cu _6_, or Zn _3_O _3_ complexes were initially formed by attaching the stable structures of two distinct complexes, namely I1 (Oxa@ CO _2_) and I1A (Oxa@ N _2_), to the most stable form of Au _6_, Cu _6_, and Zn _3_O _3_ (a planar triangle with D3h symmetry), using electron-rich positions such as N ^59, 60, 62, 63^. Harmonic vibration frequencies were also calculated at the same theoretical level to identify the obtained structures as local minima. Figure 4 displays the optimized structures for Oxa@Au _6_, Cu _6_ and Zn _3_O _3_ and for CO _2_/N _2_–Oxa@Au _6_, Cu _6_ and Zn _3_O _3_. This figure shows that in the Oxazole@cluster structure, the gold, copper, or zinc atoms situated at the apex of the octahedron form a covalent bond interaction at the O4 positions of the oxazole molecule. As seen in Figure 4, both gases are physically adsorbed, and the I1 and I1A structures of the Oxa@CO _2_ and Oxa@N _2_ complexes are moderately perturbed by the presence of the cluster. Consequently, in the structure I1@Au _6_ and I1@Cu _6_, the hydrogen bond between the oxygen O10 and H6 of the oxazole breaks, and a new hydrogen bond forms between O11 and H7 of the oxazole. The calculated MP2/AVDZ/ LanL2DZ Binding energies (BEs) of the CO _2_/N _2_–Oxa@Au _6_,Cu _6_ and Zn _3_O _3_ complexes are listed in Table 7. Our results show that metallic and metal oxide clusters favor the adsorption of CO _2_ and N _2_ gases. In hexametallic clusters, the adsorption energies (E _b_) reach a maximum with the Cu _6_ cluster, at -6.46 kcal/mol for CO _2_ and -10.25 kcal/mol for N _2_. However, the Au _6_ and Zn _3_O _3_ clusters exhibit similar binding energy values: E _b_(Au _6_@I1) = -4.58 kcal/mol and E _b_(Zn _3_O _3_@I1) = -4.56 kcal/mol for CO _2_, and E _b_(Au _6_@I1) = -2.19 kcal/mol and E _b_(Zn _3_O _3_@I1) = -2.22 kcal/mol for N _2_. This is highly advantageous as it allows for the replacement of expensive gold clusters with more cost-effective materials such as Zn _3_O _3_.

**Table 7.** MP2/AVDZ/LanL2DZ binding energies of the CO _2_/N _2_@–Oxa@Au _6_, Cu _6_ and Zn _3_O _3_ complexes (E _b_, kcal/mol).

		Au _6_	Cu _6_	Zn _3_O _3_
Complexes	I1	I1	I1	I1
Oxazole@CO _2_	-4.48	-4.58	-6.46	-4.56
Oxazole@N _2_	-1.77	-2.19	-10,25	-2.22

***Adsorption of CO _2_ and N _2_ in the metal and metal oxide surfaces (111)***

The Molecular simulation (Monte Carlo) was conducted to examine the adsorption of CO _2_ and N _2_ on Au, Cu, and ZnO (111) surfaces. The choice of Au (111) and Cu (111) surfaces is motivated by the interest in the interaction of small molecules Au (111) surface. Furthermore, adsorption on noble metals (such as Cu, Ag, and Au) is considered a promising avenue for materials in future technologies and applications ^64^, however, the selection of ZnO aims to ensure high efficiency in the low-cost adsorption of pollutants. The investigation commenced with optimizing the geometries of the Oxa@CO _2_ and Oxa@N _2_ complexes using the COMPASS II force field in the Forcite Module. Subsequently, we constructed three-layer models for each surface (Au(111), Cu(111), and ZnO(111)). In the Adsorption Locator Module, we selected one of these surfaces and added the adsorbate, either Oxa@CO _2_ or Oxa@N _2_, before running the calculations. This process was repeated for all surfaces. The optimized geometries and their corresponding adsorption energies are depicted in the Figure 5. The calculations show spontaneous physisorption of CO _2_ and N _2_. Additionally, the ZnO surface is the most favorable for N _2_ adsorption compared to CO _2_, with an adsorption energy of -151.62 kcal/mol for N _2_, while for CO _2_, it reaches -148.01 kcal/mol. However, for the two other surfaces, Cu (111) and Au (111), the adsorption of CO _2_ is more favorable than that of N2, with respective adsorption energy values of -36.97 kcal/mol and -32.88 kcal/mol for Au, and -22.27 kcal/mol and -19.52 kcal/mol for Cu (111). Overall, our calculations demonstrate that the surfaces of gold (Au), copper (Cu), and zinc oxide (ZnO) are capable of adsorbing N _2_ and CO _2_ from gas mixtures. Moreover, ZnO and Cu offer advantages in terms of adsorption capacity for both gases, without introducing additional cost-related issues.

***Sorption isotherms***

The adsorption isotherms of oxazole@CO _2_ and oxazole@N _2_ on Au (111), Cu (111), and ZnO (111) surfaces were calculated with the GCMC ^65, 66^ procedure implemented in the sorption module in BIOVIA Materials Studio. These simulations were carried out in the μVT ensemble, where the chemical potential (μ), volume (V), and temperature (T) are held constant, allowing the number of gas molecules in the system to fluctuate naturally in response to the external reservoir. All simulations were performed at 298 K over a pressure range of 1000 to 10,000 kPa. The chemical potential corresponding to each pressure point was determined using the Peng–Robinson equation of state, which accounts for non-ideal gas behavior. The Metropolis algorithm was applied to sample system configurations through stochastic insertion, deletion, translation, and rotation of molecules, ensuring ensemble consistency. Long-range electrostatic interactions between the adsorbates and the surfaces were calculated using the Ewald summation method with an energy accuracy of 0.001 kcal/mol, while van der Waals interactions were modeled using the atom-based method with a cutoff distance of 12.5 Å. Each simulation consisted of 10,000 loading steps for system equilibration and 100,000 production steps to collect statistically meaningful adsorption data. This computational approach provides detailed insight into the pressure-dependent adsorption behavior of oxazole-functionalized gases on metallic

**Figure 6.**
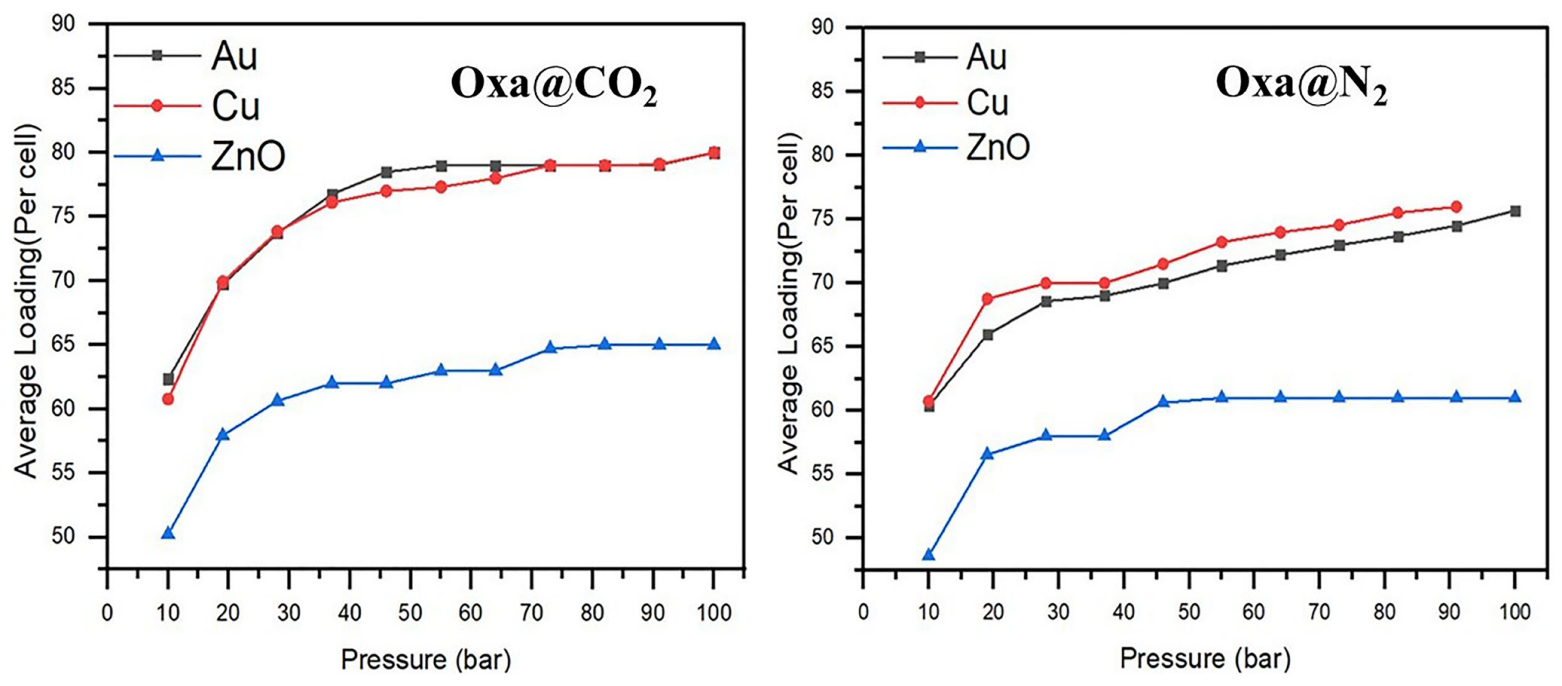
Calculated Oxa@CO _2_ and Oxa@N _2_ adsorption isotherms at 298 K on Au (111), Cu (111), and ZnO (111) surfaces.

The GCMC adsorption isotherm is a key tool for evaluating and quantifying the gas adsorption capacity on the hosts. The adsorption isotherms of Oxazole@CO _2_ and Oxazole@N _2_ on Au (111), Cu (111), and ZnO (111) surfaces, presented in Figure 6, reveal that all three surfaces generally exhibit better adsorption performance for CO _2_ compared to N _2_.

For instance, at a pressure of 19 bars, the average loading of Oxazole@CO _2_ is 57 on ZnO (111), and 69 on both Cu (111) and Au (111), whereas for Oxazole@N _2_, the values are 56.6 for ZnO (111), 68.8 for Cu (111), and 65.9 for Au (111). When the pressure increases to 91 bars, the number of CO _2_ molecules adsorbed reaches 65 on ZnO (111), and 79 on both Cu (111) and Au (111), while for Oxazole@N _2_, the values reach 61, 74.5, and 76, respectively.

In the case of Oxazole@CO _2_, the ZnO (111) surface appears to reach saturation adsorption equilibrium at a relatively high pressure, around 73 bars. In contrast, for Oxazole@N _2_, equilibrium seems to be reached earlier, around 46 bars.

Given the trend of the adsorption isotherms, it is likely that the Au (111) and Cu (111) surfaces only reach adsorption equilibrium at around 55 bars for gold and 73 bars for copper in the case of Oxazole@CO _2_. On the other hand, for Oxazole@N _2_, adsorption continues to increase significantly with rising pressure. Therefore, the Cu surface, in particular, is expected to offer even greater adsorption capacity for both CO _2_ and N _2_ at higher pressures.

## Conclusion

In summary,Our study demonstrates that oxazole has a significant adsorption capacity for both CO _2_ and N _2_, with a notably higher capacity for CO _2_ . The presence of a second gas does not affect oxazole’s ability to capture both gases simultaneously, but it may enhance adsorption through the formation of hydrogen bonds among the three systems. The addition of clusters Au _6_, Cu _6_, and Zn _3_O _3_ to the most stable structures I1 of the Oxa@CO _2_ complex and I1A of the Oxa@N _2_ complex increases the adsorption capacity for both gases, with Cu _6_ exhibiting the highest capacity compared to Au _6_ and Zn _3_O _3_. Finally, the investigation of interactions between the most stable structures of the Oxa@CO _2_ and Oxa@N _2_ complexes with the three (111) surfaces of Au, Cu, and ZnO demonstrates that gold (Au), copper (Cu), and zinc oxide (ZnO) surfaces are capable of adsorbing N _2_ and CO _2_ from gas mixtures. Moreover, Cu offer advantages in terms of adsorption capacity for both gases, without introducing additional cost-related issues.

## Ethics and consent

Ethics and consent were not required.

## Data Availability

No data are associated with this article
