# Supplementary material for: Theoretical structural study of van der Waals complexes between oxazole and atmospheric gases CO 2 and N 2 for capture applications
Source: Open Res Eur. 2025 Sep 22;5:3. Originally published 2025 Jan 8. [Version 4] doi: 10.12688/openreseurope.18925.4 (PMC12423626; doi:10.12688/openreseurope.18925.4)
Supplement: Peer review history

# Reviewer report: Reviewer response for version 4

**DOI:** 10.21956/openreseurope.23205.r60899

**Published:** 2025-09-26

**Authors:** Driss benabdallah

**Affiliations:** 1 University of Tunis El Manar, Tunis, Tunis, Tunisia; 2 University of Carthage Preparatory Institute for Engineering Studies of Nabeul (Ringgold ID: 198832), Mrezga, Nabeul, Tunisia

It's OKay

Is the study design appropriate and does the work have academic merit?

Yes

Is the work clearly and accurately presented and does it cite the current literature?

Yes

If applicable, is the statistical analysis and its interpretation appropriate?

Not applicable

Are all the source data underlying the results available to ensure full reproducibility?

No

Are the conclusions drawn adequately supported by the results?

Yes

Are sufficient details of methods and analysis provided to allow replication by others?

Yes

Reviewer Expertise:

My area of expertise is in non-reactive molecular dynamics, particularly ab initio calculations of potential energy surfaces for van der Waals systems, followed by the calculation of rotational transition cross-sections from molecular collisions, and the subsequent determination of collisional rate coefficients.

I confirm that I have read this submission and believe that I have an appropriate level of expertise to confirm that it is of an acceptable scientific standard.

# Reviewer report: Reviewer response for version 3

**DOI:** 10.21956/openreseurope.23131.r60087

**Published:** 2025-09-17

**Authors:** Driss benabdallah

**Affiliations:** 1 University of Tunis El Manar, Tunis, Tunis, Tunisia; 2 University of Carthage Preparatory Institute for Engineering Studies of Nabeul (Ringgold ID: 198832), Mrezga, Nabeul, Tunisia

The authors have carefully addressed the comments raised in the previous review. The revised manuscript shows clear improvements in clarity, consistency, and scientific accuracy.

 Notably, the consistent use of notation (“CO₂–Oxa” and “N₂–Oxa”), the inclusion of additional computational details (CCSD(T)-F12b validations), and the updated figures and tables have strengthened the overall quality of the paper. The rephrasing of sentences has also improved readability.

 I consider that the authors have satisfactorily resolved the concerns raised in my earlier report.

Is the study design appropriate and does the work have academic merit?

Yes

Is the work clearly and accurately presented and does it cite the current literature?

Yes

If applicable, is the statistical analysis and its interpretation appropriate?

Not applicable

Are all the source data underlying the results available to ensure full reproducibility?

No

Are the conclusions drawn adequately supported by the results?

Yes

Are sufficient details of methods and analysis provided to allow replication by others?

Yes

Reviewer Expertise:

My area of expertise is in non-reactive molecular dynamics, particularly ab initio calculations of potential energy surfaces for van der Waals systems, followed by the calculation of rotational transition cross-sections from molecular collisions, and the subsequent determination of collisional rate coefficients.

I confirm that I have read this submission and believe that I have an appropriate level of expertise to confirm that it is of an acceptable scientific standard.

# Reviewer report: Reviewer response for version 2

**DOI:** 10.21956/openreseurope.21961.r58175

**Published:** 2025-09-10

**Authors:** Dharamashi Rabari

**Affiliations:** 1 Ahmedabad University, Ahmedabad, Gujarat, India

The research article explores molecular interactions through ab initio calculations. The findings suggest strong selectivity for CO2 over N2. 

 It can be accepted with following minor revisions.

  

-
  - Oxazole is first introduced without abbreviation; later, you use “Oxa.” Please introduce the abbreviation at first mention, e.g., *“oxazole (Oxa)” in introduction itself *
  - The statement *“I recommend the open-source software Quantum ESPRESSO and CP2K”* can be rephrased to a neutral tone
  - Maintain uniform usage: e.g., *“Oxazole–N*₂ *”* instead of switching between *“Oxazole-N*₂ *”* and *“oxazole-N2.”*Similarly, use consistent formatting for *Oxazole–CO*₂.
  - In Figure 1, the atom is marked as “H6,” while in the text it is referred to as *“Hydrogen 6.”* Please check.
  - Figure 6: Specify the adsorption loading units used 
  - Revise “In summary,Our study” to “In summary, Our study” (spacing).
  -
    - Oxazole is first introduced without abbreviation; later, you use “Oxa.” Please introduce the abbreviation at first mention, e.g., *“oxazole (Oxa)” in introduction itself *
    - The statement *“I recommend the open-source software Quantum ESPRESSO and CP2K”* can be rephrased to a neutral tone
    - Maintain uniform usage: e.g., *“Oxazole–N*₂ *”* instead of switching between *“Oxazole-N*₂ *”* and *“oxazole-N2.”*Similarly, use consistent formatting for *Oxazole–CO*₂.
    - In Figure 1, the atom is marked as “H6,” while in the text it is referred to as *“Hydrogen 6.”* Please check.
    - Figure 6: Specify the adsorption loading units used 
    - Revise “In summary,Our study” to “In summary, Our study” (spacing).

Is the study design appropriate and does the work have academic merit?

Yes

Is the work clearly and accurately presented and does it cite the current literature?

Yes

If applicable, is the statistical analysis and its interpretation appropriate?

Yes

Are all the source data underlying the results available to ensure full reproducibility?

Yes

Are the conclusions drawn adequately supported by the results?

Yes

Are sufficient details of methods and analysis provided to allow replication by others?

Yes

Reviewer Expertise:

Computational chemistry, Molecular Simulation, Deep Eutectic Solvents Applications

I confirm that I have read this submission and believe that I have an appropriate level of expertise to confirm that it is of an acceptable scientific standard.

# Reviewer report: Reviewer response for version 2

**DOI:** 10.21956/openreseurope.21961.r57379

**Published:** 2025-08-28

**Authors:** Driss benabdallah

**Affiliations:** 1 University of Tunis El Manar, Tunis, Tunis, Tunisia; 2 University of Carthage Preparatory Institute for Engineering Studies of Nabeul (Ringgold ID: 198832), Mrezga, Nabeul, Tunisia

The manuscript is well written and easy to follow, with a clear structure and a good selection of recent references. The study uses a combination of *ab initio* MP2 calculations and Monte Carlo simulations for two-body, three-body, and surface adsorption systems. This approach is well suited to the topic and gives results of clear scientific interest, especially showing that oxazole binds more strongly to CO₂. The methods are described in enough detail to allow other researchers to repeat the work, although providing input files, Cartesian coordinates, and raw energies would make reproduction easier. No formal statistical analysis is needed, and the way the results are interpreted is reasonable. However, since there are no datasets linked to the article, sharing the source data would make the work more transparent and repeatable. The conclusions are supported by the structural, energetic, and adsorption isotherm results, and the suggested minor revisions are mainly to improve clarity, consistency, and small methodological points.

 Main Points for Revision

 1. Clarity and presentation

- Repetition error – Page 4, line ~16:
  Replace 'to estimate the adsorption isotherms to estimate the adsorption isotherms' with:
  “...performed to estimate the adsorption isotherms of oxazole@CO₂ and oxazole@N₂ on Au(111), Cu(111), and ZnO(111) surfaces.”
- Grammar – Page 4, line ~27:
  “I recommend the open-source software Quantum ESPRESSO and CP2K” → replace 'software' by 'software packages'.
- Clarity – Page 5, lines 1–3:
  Rewrite awkward sentence on structure optimization:
  “The structures of oxazole, oxazole@N₂, and oxazole@CO₂ were optimized to obtain their lowest-energy configurations using the COMPASS II force field, as implemented in the Forcite Module.”
- Paragraph restructuring – Page 6, lines 3–8:
  Rewrite for clarity:
  “This section examines the adsorption and activation of CO₂ and N₂ on small metal clusters (Au₆, Cu₆) and on the metal oxide cluster Zn₃O₃. Even-numbered clusters were chosen because they tend to be more stable and have larger HOMO–LUMO gaps, ensuring reliable structures during adsorption studies.”
- Proofreading – Final check for grammar and typographic consistency.

2. Methodological details

- Computational details – Page 5, line ~20:
  Clarify basis sets for metals and non-metals:
  “...MP2 method with aug-cc-pVDZ for non-metals and LANL2DZ ECP for metals.”
- Validation of MP2 – Suggest running a single-point CCSD(T)-F12 calculation on one representative complex (CO₂–Oxa or N₂–Oxa) to confirm MP2 accuracy.

3. Consistency and formatting

- Notation consistency – Throughout manuscript:
  Use “CO₂–Oxa” instead of “Oxa@CO₂” for clarity and ensure consistent A/B labeling (CO₂ = A, N₂ = B).
- Figure/Table consistency –
  - Table 1: bold CO₂ as for Oxazole.
  - Table 3: bold N₂, check “ER” vs “Er”.
  - Table 5: fix “Oxazole-0N₂” → “Oxazole–N₂”.
  - Figures 2A/2B: clarify I1A, I2B, etc.

Is the study design appropriate and does the work have academic merit?

Yes

Is the work clearly and accurately presented and does it cite the current literature?

Yes

If applicable, is the statistical analysis and its interpretation appropriate?

Not applicable

Are all the source data underlying the results available to ensure full reproducibility?

No

Are the conclusions drawn adequately supported by the results?

Yes

Are sufficient details of methods and analysis provided to allow replication by others?

Yes

Reviewer Expertise:

My area of expertise is in non-reactive molecular dynamics, particularly ab initio calculations of potential energy surfaces for van der Waals systems, followed by the calculation of rotational transition cross-sections from molecular collisions, and the subsequent determination of collisional rate coefficients.

I confirm that I have read this submission and believe that I have an appropriate level of expertise to confirm that it is of an acceptable scientific standard, however I have significant reservations, as outlined above.

# Reviewer report: Reviewer response for version 2

**DOI:** 10.21956/openreseurope.21961.r57251

**Published:** 2025-08-27

**Authors:** Nguyen Thi Xuan Huynh

**Affiliations:** 1 Quy Nhon University, Quy Nhon City, Vietnam

There are no comments for the revised version.

Is the study design appropriate and does the work have academic merit?

Yes

Is the work clearly and accurately presented and does it cite the current literature?

Partly

If applicable, is the statistical analysis and its interpretation appropriate?

Yes

Are all the source data underlying the results available to ensure full reproducibility?

Yes

Are the conclusions drawn adequately supported by the results?

Partly

Are sufficient details of methods and analysis provided to allow replication by others?

Yes

Reviewer Expertise:

Computational physics, Computational Physical Chemistry

I confirm that I have read this submission and believe that I have an appropriate level of expertise to confirm that it is of an acceptable scientific standard.

# Reviewer report: Reviewer response for version 1

**DOI:** 10.21956/openreseurope.20477.r50891

**Published:** 2025-03-18

**Authors:** Nguyen Thi Xuan Huynh

**Affiliations:** 1 Quy Nhon University, Quy Nhon City, Vietnam

I have some additional suggestions as follows:

 1. Check for spelling errors and wordiness in some places, and make sure it is concise and meaningful. For example, whether or not there is a comma "," or whether "the Figure ..." is used before the Figure, plural or singular, etc. There are many spelling mistakes in the manuscript, such MOF's (MOFs), labelling (labeling),...

 2. Consistent use of kcal/mol energy units for Er (relative energies) in Tables 1 and 2, consistent with the data used in the text. Unify the use of the units in kcal/mol or kcal.mol^-1...

 3. Calculations should be focused in the "Calculation details" section without needing to be explained separately in the results and discussion sections.

 4. The authors should only give important information in the Conclusion section. For example, the authors can omit the first sentence about the method.

 5. Please describe the Monte Carlo simulation method in more detail. This simulation method can quantify the gas adsorption capacity on the hosts. The authors should supplement their calculation results through adsorption isotherms.

 6. The authors need to further elucidate the interactive nature of important adsorption systems through density of states or charge density differences.

 7. Replace the figures due to blur and have to underline the captions, as shown in Figure 5.

 8. Check your references in manuscript. Note that the superscript numerals are placed after quotation marks, commas, and periods.

Is the study design appropriate and does the work have academic merit?

Yes

Is the work clearly and accurately presented and does it cite the current literature?

Partly

If applicable, is the statistical analysis and its interpretation appropriate?

Yes

Are all the source data underlying the results available to ensure full reproducibility?

Yes

Are the conclusions drawn adequately supported by the results?

Partly

Are sufficient details of methods and analysis provided to allow replication by others?

Yes

Reviewer Expertise:

Computational physics, Computational Physical Chemistry

I confirm that I have read this submission and believe that I have an appropriate level of expertise to confirm that it is of an acceptable scientific standard, however I have significant reservations, as outlined above.

# Response

**Published:** 2025-04-25

**Authors:** samira dalbouha

**Affiliations:** chimie, Universite Ibn Zohr Faculte des Sciences Agadir, Agadir, Souss-Massa-Draa, Morocco

We sincerely thank the reviewer for their valuable comments on our manuscript. These constructive remarks and suggestions have significantly contributed to the improvement of our work. In response to these comments, we have prepared a revised and enhanced version of the manuscript. *(The reviewer's comments are presented in italics.)* ***Comment-1 :*** *Check for spelling errors and wordiness in some places, and make sure it is concise and meaningful. For example, whether or not there is a comma "," or whether "the Figure ..." is used before the Figure, plural or singular, etc. There are many spelling mistakes in the manuscript, such MOF's (MOFs), labelling (labeling),...* ***Reply-1 :*** We have carefully reviewed the manuscript and addressed the spelling errors and wordiness issues as suggested. Proper punctuation has been ensured, singular/plural usage corrected, and references to figures standardized. Additionally, the specific spelling mistakes mentioned, such as *MOF’s* (corrected to *MOFs*) and *labelling* (corrected to *labeling*), have been revised accordingly. ***Comment-2 :*** *Consistent use of kcal/mol energy units for Er (relative energies) in Tables 1 and 2, consistent with the data used in the text. Unify the use of the units in kcal/mol or kcal.mol^-1...* ***Reply-2 :*** We have taken your suggestion into account and have unified the use of energy units in Tables 1 and 2. The relative energy values (Er) are now consistently expressed in kcal/mol, in line with the data used in the text. ***Comment-3 :*** *Calculations should be focused in the "Calculation details" section without needing to be explained separately in the results and discussion sections.* ***Reply-3 :*** We have taken your suggestion into account and moved the calculation details to the "Calculation Details" section. ***Comment-4 :*** *The authors should only give important information in the Conclusion section. For example, the authors can omit the first sentence about the method.* ***Reply-4 :*** We have revised the Conclusion section to include only the essential information, omitting the first sentence regarding the method, as suggested, to make it more concise and focused on the key results. ***Comment-5 :*** *Please describe the Monte Carlo simulation method in more detail. This simulation method can quantify the gas adsorption capacity on the hosts. The authors should supplement their calculation results through adsorption isotherms.* ***Rpply-5 :*** We have taken your suggestion into account and added a detailed description of the Monte Carlo simulation method in the article. Additionally, a new section titled "sorption Isotherms" has been included, along with Figure 6, which illustrates the adsorption isotherms. These additions complement our calculation results and allow for the quantification of the gas adsorption capacity on the hosts. ***Comment-6 :*** *The authors need to further elucidate the interactive nature of important adsorption systems through density of states or charge density differences.* ***Reply-6 :*** We thank the reviewer for this insightful comment. We would like to clarify that this article is part of a series of studies conducted within the framework of our research project on the adsorption of pollutant gases. A detailed investigation of the interactive nature of the adsorption systems, particularly through density of states (DOS) and charge density difference analyses, will be presented in a complementary article currently in preparation, which will specifically focus on the electronic aspects of the systems studied here. ***Comment-7 :*** *Replace the figures due to blur and have to underline the captions, as shown in Figure 5.* ***Reply-7:*** We have taken your comment into account. All the blurred figures have been replaced with higher-quality versions, specifically Figures 1, 2, 3A, 3B, and 5. ***Comment-8 :*** *Check your references in manuscript. Note that the superscript numerals are placed after quotation marks, commas, and periods.* ***Reply-8 :*** We have taken your comment into account and revised the manuscript accordingly. The superscript reference numerals have been repositioned to appear after quotation marks, commas, and periods, as required.
